# First report of *Lasiodiplodia pseudotheobromae* keratitis susceptible to voriconazole in an Indian mango grower

**DOI:** 10.1099/acmi.0.000055

**Published:** 2019-08-16

**Authors:** Hari Pankaj Vanam, Mohammed Ather, K. S. Madhura, Shivprakash Mandya Rudramurthy

**Affiliations:** ^1^ Mycology Division, Department of Microbiology, Bhaskar Medical College and General Hospital, Bhaskar Nagar, Yenkapally, Moinabad, R.R. District, Telangana 500 075, India; ^2^ Department of Ophthalmology, Bhaskar Medical College and General Hospital, Bhaskar Nagar, Yenkapally, Moinabad, R.R. District, Telangana 500 075, India; ^3^ Department of Medical Microbiology, Postgraduate Institute of Medical Education and Research, Chandigarh 160012, India

**Keywords:** ITS sequencing, phytopathogenic fungi, fungal keratitis, topical voriconazole, phylogenetic analysis, *Lasiodiplodia pseudotheobromae*

## Abstract

The family *Botriosphaeriacea* comprises cosmopolitan endophytic fungi and many of the genera have frequently been implicated in causing human infections, including subcutaneous infection, sinusitis, invasive mycoses and keratitis. Among them, the genus *Lasiodiplodia*, which contains >30 species, is grouped as coelomycetous fungi with prototype species *Lasiodiplodia theobromae* as an important cause of keratitis. Several cryptic species morphologically resembling *L. theobromae* exist, one of which is *Lasiodiplodia pseudotheobromae*. We present a rare case of mycotic keratitis in an Indian mango grower following penetrating trauma to the left eye. Direct microscopy revealed retractile hyphal elements, and fungal cultures yielded a dematiaceous mould which was confirmed by sequencing as *L. pseudotheobromae*. Antifungal susceptibility testing revealed low MICs to amphotericin B and voriconazole and increased MICs to itraconazole and posaconazole. This is the first report of phytopathogenic *L. pseudotheobromae* keratitis, successfully treated with 2 % voriconazole without keratoplasty.

## Introduction

Fungal keratitis (FK) or keratomycosis is an infection of the cornea caused by a multitude of fungal genera. In tropical countries, antecedent ocular trauma is a significant predisposing factor for fungal invasion of the ocular surface. It generally manifests as slow progressive corneal ulcers. FK comprises 40 % of corneal ulcers and must be differentiated from bacterial, viral and parasitic infections of the eye [1]. The global burden of FK is unprecedented and represents an important cause of blindness following ocular trauma and corneal ulceration [1]. Established fungal aetiologies causing FK include *Aspergillus* species, *Fusarium* species, *Curvularia* species, *Scedosporium apiospermum* and *Paecilomyces*; *Aspergillus* species are the predominant agents of FK following ocular trauma [1]. Furthermore, there is a geographical variation in the prevalence of specific agents causing FK worldwide, especially in developing countries like India [1]. The family *Botriosphaeriacea* are cosmopolitan endophytic fungi and many of the genera are established plant pathogens [2]. Notable genera of *Botryospaeriales* frequently implicated in causing human infections such as subcutaneous infection, sinusitis, invasive mycoses and keratitis are *Lasiodiplodia*, *Macrophomina* and *Neoscytalidium* [2, 3]. The genus *Lasiodiplodia* comprises dematiaceous pycnidial coelomycetous fungi classified within the subphylum Pezizomycotina of Ascomycota [2, 3]. The prototype species of *Lasiodiplodia* is *Lasiodiplodia theobromae*, an important cause of keratitis accounting for over 50 cases of corneal ulcers worldwide and which are reportedly refractory to antifungal treatments [4]. Morphological and phylogenetic studies have revealed several reports of cryptic speciation in *L. theobromae*. Presently there are >30 species in the genus *Lasiodiplodia* which share close genetic homology including the cryptic species *Lasiodiplodia pseudotheobromae* [5, 6]. Both *L. theobromae* and *L. pseudotheobromae* are established plant pathogens causing post-harvest fungal disease of grape wine yards, mango and citrus plantations, resulting in extensive economic losses worldwide [7, 8].

We report the first case of phytopathogenic *L. pseudotheobromae* causing keratitis in India. This infection was susceptible to voriconazole, and was successfully treated with 2 % voriconazole without keratoplasty.

## Case report

**Fig. 1.**
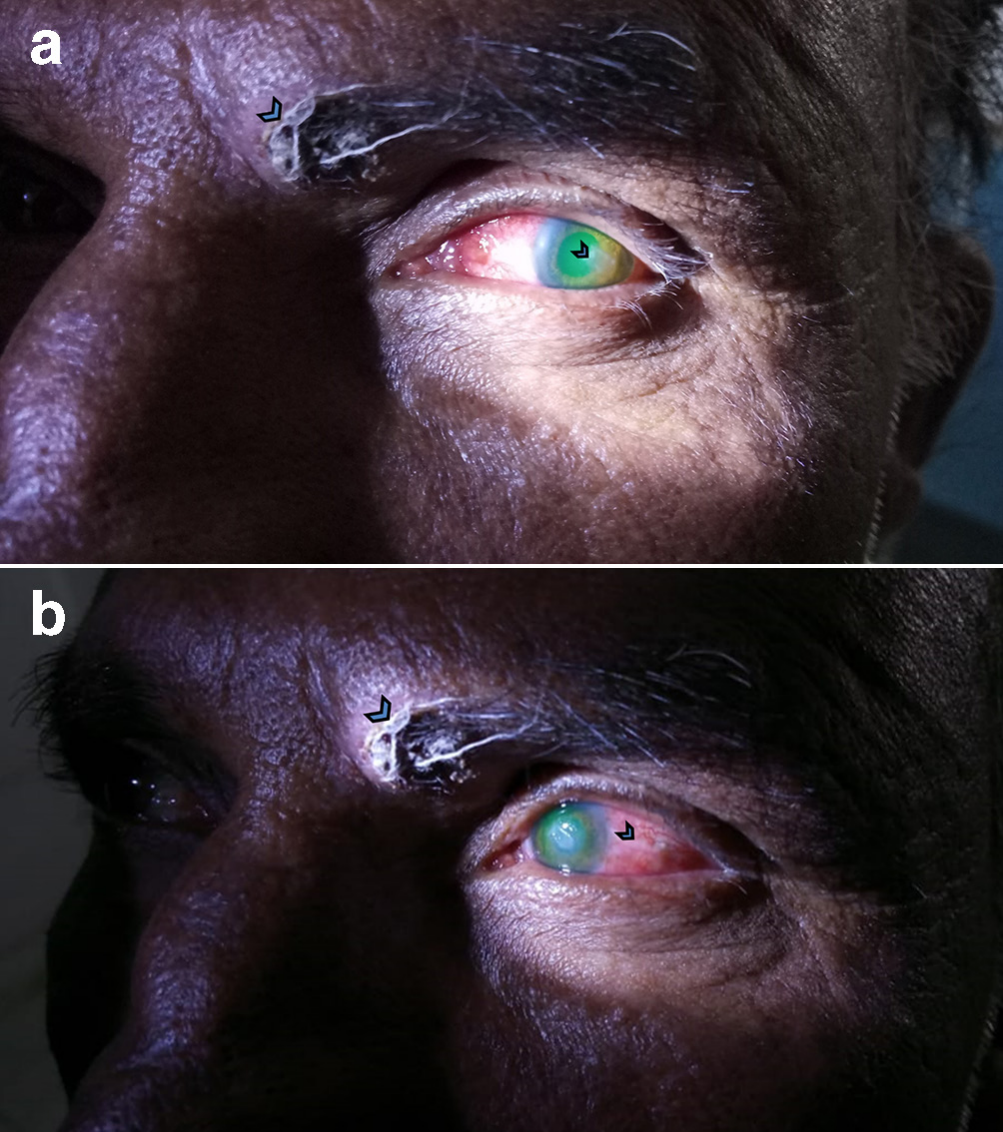
Ophthalmic examination revealing a corneal ulcer and circumcorneal congestion. (a) A 1×2 mm laceration over the left eyebrow (arrowhead) on the medial aspect and a dirty-white corneal ulcer of 2×3 mm (arrowhead). (b) Laceration wound on the left eyebrow (arrowhead) and circumcorneal congestion (arrowhead).

A 50-year-old male farmer who was a caretaker of mango groves presented to the ophthalmology outpatient department (OPD) with complaints of pain, redness, watering, photophobia and diminished vision in the left eye over 4 days (day 8). He had sustained an injury to the left eyebrow and traumatic inoculation with a tree branch into the left eye 7 days previously while working in mango groves (day 0), for which he had repeatedly applied a non-sterile limestone powder. On examination, a small 1×2 mm laceration was found over the left eyebrow on the medial aspect. Ocular examination revealed circumcorneal congestion and a dry-looking corneal ulcer of 2×3 mm, dirty-white in colour, with feathery margins, situated at the 3 o’clock position, 2 mm from the limbus covering a part of the pupil. The ulcer was extending up to the superficial layers of the stroma and was covered with minimal slough (Fig. 1a, b). The anterior chamber was quiet, pupils briskly reacting to light and the lens showed early nuclear sclerosis. His visual acuity in the left eye was counting fingers from 3 m. General and systemic examination of the patient was unremarkable. There was no history of any concomitant infections.

Corneal scrapings revealed fungal hyphae on direct potassium hydroxide (KOH) mount. The patient was started on a topical suspension of 5 % natamycin hourly along with 0.5 % moxifloxacin eye drops six times daily, and 1 % cyclopentolate drops three times daily. His follow-up visit after 4 days showed only symptomatic relief, but the ulcer was 3×3 mm in size and showed no signs of response. He was prescribed to continue the same treatment and report after another 4 days.

The patient showed no significant response to natamycin and moxifloxacin therapy even after 7 days (day 11), and at this juncture a fungal culture isolated *L. pseudotheobromae*. The treatment was revised to voriconazole eye drops twice hourly by discontinuing moxifloxacin and natamycin eye drops. A solution of sterile 1 % voriconazole eye drops (10 mg ml^−1^) was prepared by using commercially available Inj. Voriconazole powder (200 mg) reconstituted in 19 ml of sterile water to give 20 ml of a 10 mg ml^−1^ voriconazole solution [9]. *In vitro* antifungal susceptibility testing (AFST) was performed using a broth microdilution (BMD) method which revealed a low MIC to voriconazole, which confirmed the initiated treatment. The patient was instructed to use the drops twice hourly and was called for a follow-up after 1 week (day 18). At the end of the 14th day (day 26), after successful treatment of 2 % voriconazole drops, the eye showed remarkable improvement, with a decrease in signs of inflammation and slight visual improvement. A fresh solution was prepared once every 2 weeks. He was advised to continue the same therapy six times daily and was suggested to attend regular follow-ups. At the end of 8 weeks (day 68) of treatment, the eye looked quiet, with a macular scar measuring 2×3 mm covering it, and his visual acuity had improved (6/36 up to 6/18).

## Microbiological investigations

Corneal scrapings were collected using a surgical scalpel blade no. 15. Scrapings were subjected to 20 % KOH mount and a Gram stain. The rest of the specimen was inoculated on blood agar, and two tubes of Sabouraud’s dextrose agar (SDA) with 0.005 g chloramphenicol (HiMedia), one incubated in ambient air at 28 °C in a BOD (Biological Oxygen Demand/Biochemical Oxygen Demand) incubator and the other in an incubator at 37 °C.

### Direct microscopy and morphological identification of the isolate

**Fig. 2.**
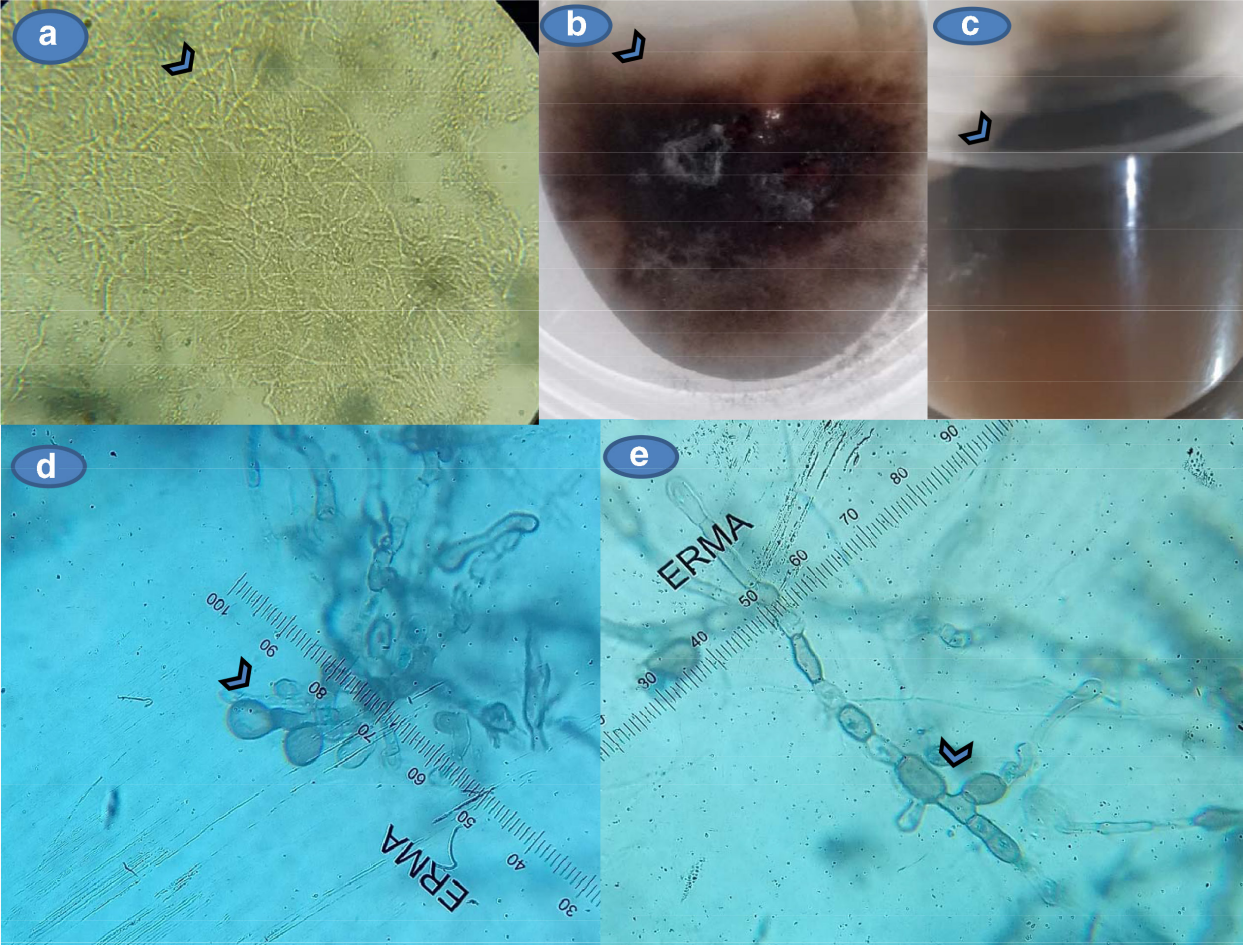
Direct microscopy and morphological features of *Lasidodiplodia pseudotheobromae* on fungal culture. (a) Extensive refractile hyphae on KOH mount, which are branching and filamentous (arrowheads). (b) Mouse grey to black cottony surface with moderate aerial mycelium at the periphery (arrowhead). (c) Black colour of the reverse. (d-e). LPCB mount of a 3-week-old culture on SDA showing sterile subhyaline to dematiaceous hyphae that are fragmented (arrowhead); conidiophores are subhyaline to dematiaceous and often septate.

A 20 % KOH mount of the corneal scrapings revealed extensive refractile hyphae that were hyaline to subhyaline, septate, branching and filamentous, suggesting a mould infection (Fig. 2a). No bacteria were seen on Gram staining. Colonies on SDA started growing after 72 h and these were initially floccose, with a white to greyish surface and greyish reverse. No visible growth was seen on blood agar after 1 week of incubation. At the end of 3 weeks of incubation, the colonies turned mouse grey to black, were floccose showing sparse aerial mycelium and the reverse was black (Fig. 2b–c,). Lacto phenol cotton blue (LPCB) mounts at the end of the 3-week period of incubation on SDA at 28 °C showed sterile subhyaline hyphae that were fragmented. Conidiophores were subhyaline to dematiaceous and often septate. Conidiogenous cells were subhyaline simple, cylindrical and holoblastic (Fig. 2d, e). Based on the above morphological findings, the isolate was tentatively reported as a dematiaceous mould. Due to the poor sporulation and pleomorphic nature of the isolate, it was deposited in the National Culture Collection of Pathogenic Fungi (NCCPF), Postgraduate Institute of Medical Education and Research (PGIMER), Chandigarh, India, for molecular confirmation with a submission ID IL_3183 (Myc_400).

### Confirmation of *L. pseudotheobromae* by molecular identification

Molecular identification of the culture was performed at the NCCPF, PGIMER, Chandigarh, India, by sequencing the ITS (internal transcribed spacer) region of the rDNA using universal primers ITS1 and ITS2. *L. pseudotheobromae* was confirmed by comparing the sequence with the ITS sequence database from the Centraalbureau voor Schimmelcultures (CBS), Utrecht, the Netherlands, International Society of Human and Animal Mycology (ISHAM) ITS database, and using the National Centre for Biotechnology Information (NCBI) nblast tool, respectively. The ITS sequence of the isolate showed 100 % similarity to *L. pseudotheobromae* CBS 116459^T^ along with other deposited *L. pseudotheobromae* ITS sequences from the NCBI, ISHAM and CBS ITS databases. The sequence was deposited in the GenBank ITS database and published in the NCBI database on 22 September 2018 under accession number MH938077.1 and name *L. pseudotheobromae* strain IL3183_Myc_400 (available in the online version of this article).

### Phylogenetic analysis

The deposited ITS sequence from the present case was compared with 28 other ITS sequences of type strains retrieved from the NCBI GenBank ITS database (https://blast.ncbi.nlm.nih.gov/Blast.cgi), ITS database from CBS (‘www.westerdijkinstitute.nl/medical/’) and ISHAM ITS database (http://its.mycologylab.org/BioloMICSSequences.aspx?expandparm=f&file=ALL), respectively (Table 1).

**Table 1.** List of reference culture collection strains and GenBank ITS sequence accession numbers included in the phylogenetic study (blue colour highlights the present submission)

**Species**	**Culture/strain**	**Accession number**	**Origin**
L.*L. pseudotheobromae (Vanam Et al. India)*	IL3183/M-400	MH938077.1	NCBI, GenBank ITS database
*L. pseudotheobromae*	CPATU01	KX171632.1	NCBI, GenBank ITS database
*L. pseudotheobromae*	CBS116459	KF766192.1	NCBI, GenBank ITS database
*L. pseudotheobromae*	CMM3999	JX464075.1	NCBI, GenBank ITS database
*L. pseudotheobromae ITS Type material*	CBS16459	NR_111264.1	NCBI, GenBank ITS database
*L. subglobosa*	CMM3872	KF234558.1	NCBI, GenBank ITS database
*L. parva*	CBS456.78	NR_111265.1	NCBI, GenBank ITS database
*L. theobromae*	CBS112874	EF622075.1	ISHAM and NCBI, GenBank ITS database
*L. mahajangana*	CBS124925	MH863425.1	NCBI, GenBank ITS database
*L. gonubiensis*	CBS115812	DQ458892.1	NCBI, GenBank ITS database
*L. lignicola*	MFLUCC 11–0435	JX646797.1	NCBI, GenBank ITS database
*L. citricola*	IRAN1522C	GU945354.1	NCBI, GenBank ITS database
*L. laeliocattleyae*	CBS167.28	NR_147364.1	NCBI, GenBank ITS database
*L. egyptiacae*	CBS130992	NR_120002.1	NCBI, GenBank ITS database
*L. venezuelensis*	WAC12539	DQ103547.1	NCBI, GenBank ITS database
*L. brasiliensis*	LAYAP1	KU507473.1	NCBI, GenBank ITS database
*L. mediterranea*	CBS137783	NR_147352.1	NCBI, GenBank ITS database
*L. pyriformis*	CBS121770	NR_136993.1	NCBI, GenBank ITS database
*L. hyalina*	CGMCC 3.17975	KX499879.1	NCBI, GenBank ITS database
*L. missouriana*	CBS128311	NR_145222.1	NCBI, GenBank ITS database
*L. rubropurpurea*	WAC12536	DQ103554.1	NCBI, GenBank ITS database
*L. macrospora*	CMM3833	KF234557.1	NCBI, GenBank ITS database
*L. rassispora*	CMW13488	DQ103552.1	NCBI, GenBank ITS database
*Diplodia cupressi*	CBS 168.87	KF766157.1	NCBI, GenBank ITS database
*Diplodia cajani*	CBS214.50	MH856592.1	NCBI, GenBank ITS database
*Diplodia corticola*	CBS112549	KF766156.1	NCBI, GenBank ITS database
*Diplodia tsugae*	CBS418.64	DQ458888.1	NCBI, GenBank ITS database
*Botryosphaeria dothidea-*	CBS115476	KF766151.1	NCBI, GenBank ITS database
*Neofusicoccum luteum*	CBS110299	AY259091.1	NCBI, GenBank ITS database

**Fig. 3.**
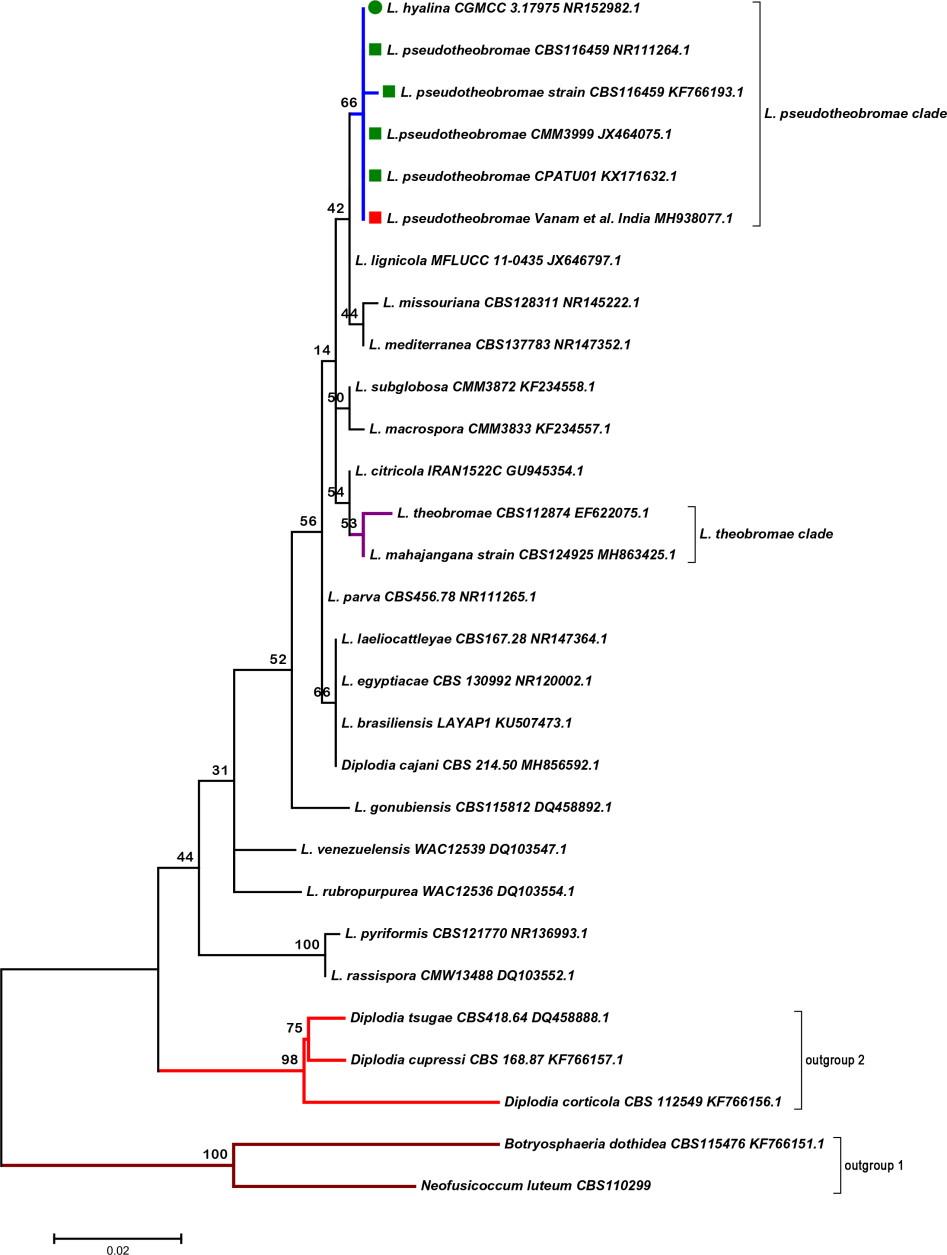
Phylogenetic tree based on ITS sequences using the ML method. The evolutionary history of 29 ITS sequences was inferred by using the ML method based on the GTR model and phylogenetic reconstruction was done automatically by applying the Neighbor-Join and BioNJ algorithms to a matrix of pairwise distances estimated using the Maximum Composite Likelihood (MCL) approach, and then selecting the topology with superior log likelihood value with a bootstrap consensus inferred from 2000 replicates. A discrete Gamma distribution was used to model evolutionary rate differences among sites [five categories (+G, parameter=0.1691)]. The tree is drawn to scale, with branch lengths measured in the number of substitutions per site [10, 11].

Alignment of multiple ITS sequences was done using the muscle (multiple sequence comparison by log-expectation) program within the mega7 software (Molecular Evolutionary Genetics Analysis: Version 7) [10, 11]. Evolutionary and phylogenetic analyses via the maximum-likelihood (ML) method were conducted using mega7 [11]. The dataset comprised a total of 29 ITS sequences retrieved from GenBank (Fig. S1). There were 19 different species of *Lasiodiplodia*, including five ITS sequences of *L. pseudotheobromae* (present submission marked as a red square) and *Diplodia cajani*. Outgroup 1 included *Neofusicoccum luteum* and *Botryospaeria dothidea*, and outgroup group 2 included three species of *Diplopia* (Fig. 3). The tree was compressed at nodes to represent genera. The tips of the external nodes represented the present species, of the internal node represented a hypothetical ancestor, and the branches connected the nodes. All positions containing gaps and missing data were eliminated. The evolutionary history was inferred by using the ML method based on the General Time Reversible (GTR) model. The internal node (blue colour) representing the clade of *L. pseudotheobromae* showed the present submission *L. pseudotheobromae* Vanam *et al*. positioned closely to the type strain ITS material of *L. pseudotheobromae* (green squares) along with *L. hyalina* (green button) (Fig. 3).

**Fig. 4.**
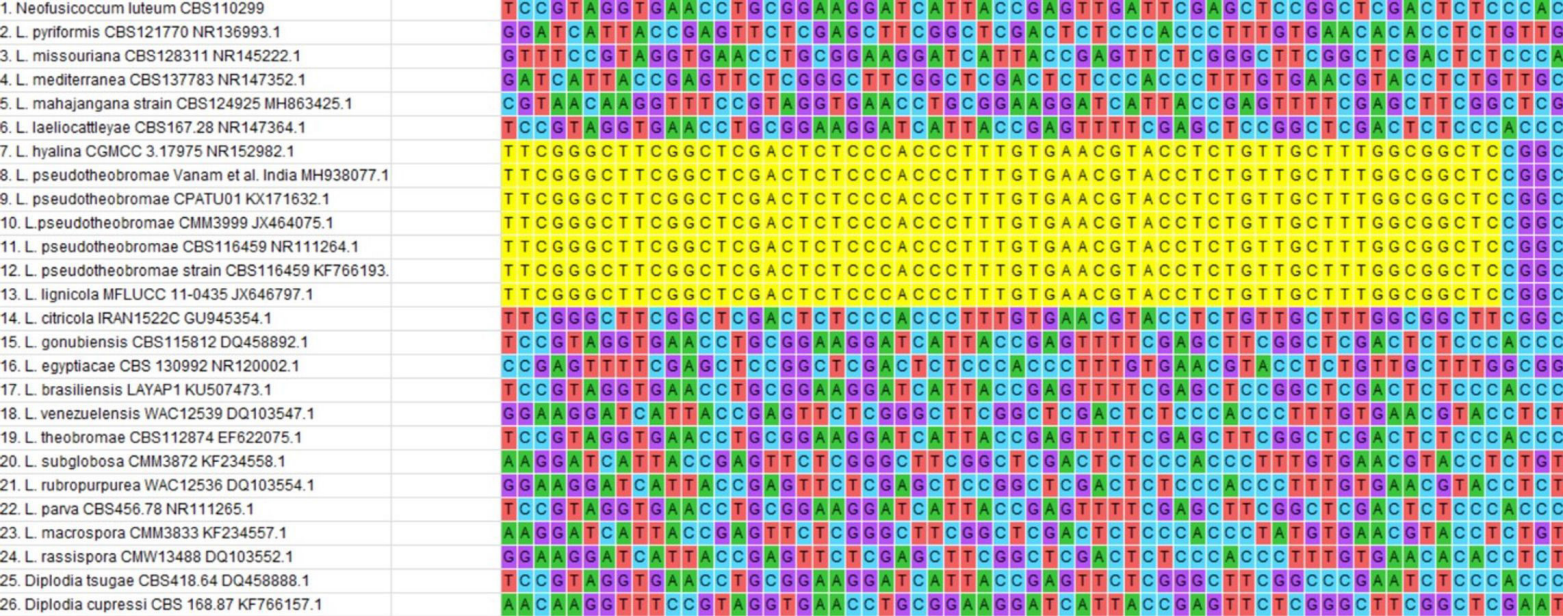
Nucleotide alignment of ITS sequences showing highly conserved regions of *L. pseudotheobromae* (highlighted in yellow).

The present sequence showed no divergence when compared with other type strain ITS material of *L. pseudotheobromae* and was positioned closely in the phylogenetic tree. There was a highly conserved region of 61 nt bases, ‘TTCGGGCTTCGGCTCGACTCTCCCACCCTTTGTGAACGTACCTCTGTTGCTTTGGCGGCTC’, seen only in *L. pseudotheobromae* and *L. hyaline* and this in turn was negative in 17 other species of *Lasiodiplodia* analysed in the present study, along with *L. theobromae* (Fig. 4). The above phylogenetic tree and evolutionary divergence analyses confirmed the isolate as representing *L. pseudotheobromae*.

### *In vitro* antifungal susceptibility testing (AFST)

*In vitro* AFST of *L. pseudotheobromae* for four-antimycotic drugs, amphotericin B (AmB), voriconazole (VOR), itraconazole (ITR) and posaconazole (POS), was performed at NCCPF, PGIMER, Chandigarh, India, using the standardized BMD method according to the Clinical Laboratory Standards International (CLSI) document M38A2 [12]. Reagent-grade antifungal powders (Sigma-Aldrich) were used. The fungal inoculum was prepared using RPMI 1640 (HiMedia) with l-glutamine and without bicarbonate and 0.165 M MOPS (Sigma-Aldrich), and was used for diluting the antimycotic drugs with pH adjusted at 7.0. The quality control strains *Candida krusei* ATCC 6258, *Aspergillus ﬂavus* ATCC 20430 and *Candida parapsilosis* ATCC 22019 were included in the protocol according to the CLSI M38-A2 document [12]. Preparation of inoculum and dilutions was performed as described by Vanam *et al*. and Rudramurthy *et al*. [13, 14]. Table 2 shows the dilution range of antimycotic agents tested in the *in vitro* BMD method used and the results of *in vitro* AFST of *L. pseudotheobromae* (IL3183_Myc 400).

**Table 2.** *In vitro* AFST results of *L. pseudotheobromae* from an Indian mango grower revealed low MIC for AmB and VOR and increased MICs for ITR and POS

**Antimycotic agent tested**	**Dilution range (µg ml^−1^)**	**MIC (µg ml^−1^)**
Amphotericin B	0.0078–4	1
Voriconazole	0.016–16	2
Itraconazole	0.016–16	16
Posaconazole	0.016–16	16

## Discussion

Morphological features such as pycnidia, paraphyses, the shape of conidia, longitudinal striations on mature conidia, etc., are widely used in distinguishing different species of *Lasiodiplodia* and other genera of *Botryosphaeriaceae*, although phylogenetically all the species of *Lasiodiplodia* exhibit less than 1 % ITS sequence divergence. Even though morphological features guide in the identification of species of *Lasiodiplodia*, the recently described *L. hyalina* sp. nov. produced only asexual forms and 10 % of the conidia became dematiaceous and predominately remained hyaline even after 3 months of culture, thus posing challenges in the identification of *Lasiodiplodia* species based on morphological features alone [15].

Only four teleomorphic species of *Lasiodiplodia* have been described, namely *L. theobromae*, *L. pseudotheobromae*, *L. lignicola* and *L. gonubiensis*, and the anamorphic–teleomorphic connection of *L. theobromae* has not yet been conclusively proven [16]. The important morphological features of *L. pseudotheobromae* are paraphyses which are hyaline, cylindrical, mostly aseptate, up to 58 µm long and 3–4 µm wide. Conidiogenous cells are hyaline, thin-walled, smooth, cylindrical, holoblastic and slightly swollen at the base, one of the features which differentiates it from *L. theobromae*. Mature conidia are ellipsoidal (apex and base are rounded), widest at the middle, thick-walled and measure 23.5–32×14–18 µm; in contrast, *L. theobromae* has smaller conidia that taper to a truncate base. Conidia are initially hyaline and aseptate for a long time, becoming one-septate and dark brown after they are released from the conidiomata, with melanin deposits on the inner surface of the wall arranged longitudinally, giving a striated appearance to the conidia. On potato dextrose agar (PDA) at 35 °C, it produces a pink pigment and also grows at 10 °C [2]. Therefore, the morphologies of *Lasiodiplodia* species show a significant degree of variation and hence identification of species without DNA sequence comparisons is inconclusive [16]. The present isolate from keratitis failed to sporulate, and other morphological features used to distinguish species of *Lasiodiplodia* were not perceptible even after 3 weeks of incubation except a pink pigment on incubation at 35 °C. The incorporation of a sporulation medium such as 2 % water agar is not routinely used in the diagnostic workup and it was not performed for the present isolate. Due to non-sporulation and pleomorphic structures, the isolate was submitted to the national reference laboratory for species confirmation using ITS sequencing, where the isolate was confirmed as representing *L. pseudotheobromae*.

There is geographical variation in the habits used by various species of *Lasiodiplodia*. Reported studies show that these are most common in the tropics and subtropics [17]. Seven species, including *L. theobromae* and *L. pseudotheobromae*, were reported as causing mango rot and present as a latent-endophyte infection [7, 8]. Pathogenicity studies of *Lasiodiplodia* species in plants revealed similarities in these two species, as both of them caused similar fruit rot symptoms but with different levels of severity (some *L. pseudotheobromae* showed more severe fruit rotting than *L. theobromae*). Interestingly, non-pathogenic isolates of both species were also identified [7]. Trauma sustained during the mango post-harvest season was likely to be a predisposing factor for the patient as he works in mango groves and had a positive history of penetrating trauma by a branch of a mango tree to the left eyebrow and small splinters of the same had penetrated the corneal stroma. The suspected source of the fungi could be from the branch of the mango plant; upon eliciting a history, the patient described the branch as being dark and necrotic in appearance and a sharp dust emanated during the injury which in turn fell in the left eye. No attempt to take a specimen from the mango grove was made for logistical reasons.

*In vitro* AFST data of *Lasiodiplodia* species implicated in causing human infections are few. In the present case, AFST of *L. pseudotheobromae* IL3183_Myc_400 revealed an increased MIC (16 µg ml^−1^) for ITR. This is in agreement with a recent study from northern India by Singh *et al*. [18], wherein similar *in vitro* susceptibility patterns to ITR with increased MICs (16 µg ml^−1^) for both *L. theobromae* (VPCI843P14) and *L. parva* (VPCI259P16) were reported [18]. Furthermore, increased MICs (16 µg ml^−1^) for POS on the present *L. pseudotheobromae* strain compare with an MIC of 8 µg ml^−1^ on *L. theobromae* (VPCI843P14) and *L. parva* (VPCI259P16) [18]. Results from the present study are in concordance with the low MICs of AmB and VOR (<2 µg ml^−1^) on *L. pseudotheobromae,* which were comparable with those of other species of *Lasiodiplodia* and hence can be considered as the most potent antimycotic agents against *Lasiodiploida* infections in humans.

Even though the diagnosis of FK is straightforward, it is often challenging because a multitude of aetiologies are involved, including unusual species such as *Lasiodiplodia* following a predisposing factor like penetrating trauma of the eye. Initiation of appropriate treatment without delay will prevent further invasion of the fungi into deeper structures and prevent complications that could threaten the vision of the patient [1]. Although natamycin is an approved first-line agent in the treatment of FK, it is not a preferred choice due to its inability to cover other filamentous fungi or infections resulting in deep stromal invasion [1]. Voriconazole, which has minimal toxicity, is a good alternative for the treatment of FK caused by a variety of filamentous fungi; some cases of *L. theobromae* keratitis have been successfully treated with topical voriconazole, resulting in good visual acuity [1, 4, 19].

Early diagnosis of fungal elements via direct microscopy and close liaison with a mycology laboratory regarding the rare mould infection prompted early initiation of voriconazole treatment. The patient responded well to voriconazole eye drops. No systemic antifungals were given. The ulcer healed with a thin macular corneal scar without the need for keratoplasty and with useful visual acuity.

## Supplementary Data

Supplementary File 1
