# Supplementary material for: First report of Lasiodiplodia pseudotheobromae keratitis susceptible to voriconazole in an Indian mango grower
Source: Access Microbiol. 2019 Aug 16;1(6):e000055. doi: 10.1099/acmi.0.000055 (PMC7470297; doi:10.1099/acmi.0.000055)

NCBI GenBank Accession no. MH938077.1

GenBank

Lasiodiplodia pseudotheobromae cultivar Hari Pankaj Vanam internal transcribed spacer 1, partial sequence; 5.8S ribosomal RNA gene and internal transcribed spacer 2, complete sequence; and large subunit ribosomal RNA gene, partial sequence

GenBank: MH938077.1

FASTA Graphics

Go to:

```
LOCUS       MH938077                 558 bp    DNA     linear   PLN 27-SEP-2018
DEFINITION  Lasiodiplodia pseudotheobromae cultivar Hari Pankaj Vanam internal
            transcribed spacer 1, partial sequence; 5.8S ribosomal RNA gene and
            internal transcribed spacer 2, complete sequence; and large subunit
            ribosomal RNA gene, partial sequence.
ACCESSION   MH938077
VERSION     MH938077.1
KEYWORDS    .
SOURCE      Lasiodiplodia pseudotheobromae
  ORGANISM  Lasiodiplodia pseudotheobromae
            Eukaryota; Fungi; Dikarya; Ascomycota; Pezizomycotina;
            Dothideomycetes; Dothideomycetes incertae sedis; Botryosphaeriales;
            Botryosphaeriaceae; Lasiodiplodia.
REFERENCE   1  (bases 1 to 558)
  AUTHORS   Vanam,H.P.
  TITLE     Lasiodiplodia pseudotheobromae rare cause of Keratitis
  JOURNAL   Unpublished
REFERENCE   2  (bases 1 to 558)
  AUTHORS   Vanam,H.P.
  TITLE     Direct Submission
  JOURNAL   Submitted (22-SEP-2018) Microbiology, Bhaskar Medical College and
            General Hospital, Moinabad, Hyderabad, Telangana 500075, India
COMMENT     ##Assembly-Data-START##
            Sequencing Technology :: Sanger dideoxy sequencing
            ##Assembly-Data-END##
FEATURES             Location/Qualifiers
     source          1..558
                     /organism="Lasiodiplodia pseudotheobromae"
                     /mol_type="genomic DNA"
                     /cultivar="Hari Pankaj Vanam"
                     /isolation_source="Corneal Scrapping"
                     /db_xref="taxon:466954"
                     /country="India"
     misc_RNA        <1..>558
                     /note="contains internal transcribed spacer 1, 5.8S
                     ribosomal RNA, internal transcribed spacer 2, and large
                     subunit ribosomal RNA"
ORIGIN
        1 aaaaaaaaaa tcaaaaaaaa ctttcctacg acataagaac attaccgagt tttcgggctt
       61 cggctcgact ctcccaccct ttgtgaacgt acctctgttg ctttggcggc tccggccgcc
      121 aaaggacctc caaactccag tcagtaaacg cagacgtctg ataaacaagt taataaacta
      181 aaactttcaa caacggatct cttggttctg gcatcgatga agaacgcagc gaaatgcgat
      241 aagtaatgtg aattgcagaa ttcagtgaat catcgaatct ttgaacgcac attgcgcccc
      301 ttggtattcc gggggcatg cctgttcgag cgtcattaca accctcaagc tctgcttgga
      361 attgggcacc gtcctcactg cggacgcgcc tcaaagacct cggcggtggc tgttcagccc
      421 tcaagcgtag tagaatacac ctcgctttgg agtggttggc gtcgcccgcc ggacgaacct
      481 tctgaacttt tctcaaggtt gacctcggat caggtaggga tacccgctga acttaagcat
      541 atcaataagc ggaggaaa
//
```

Phylogenetic analysis:

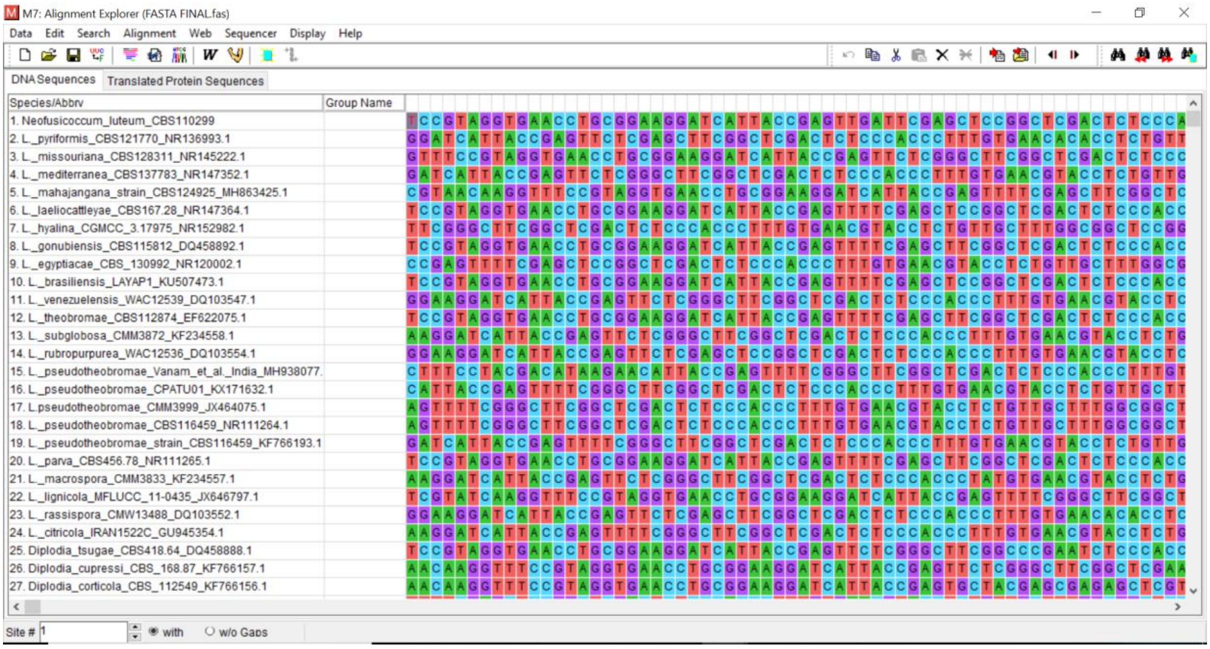

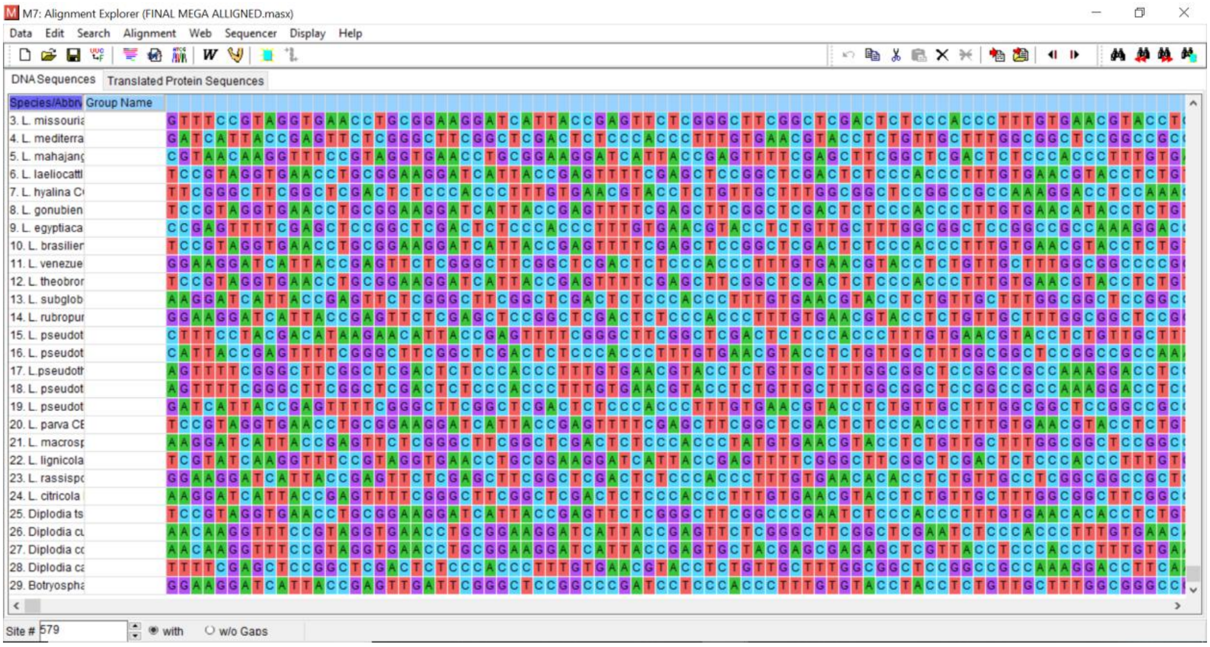

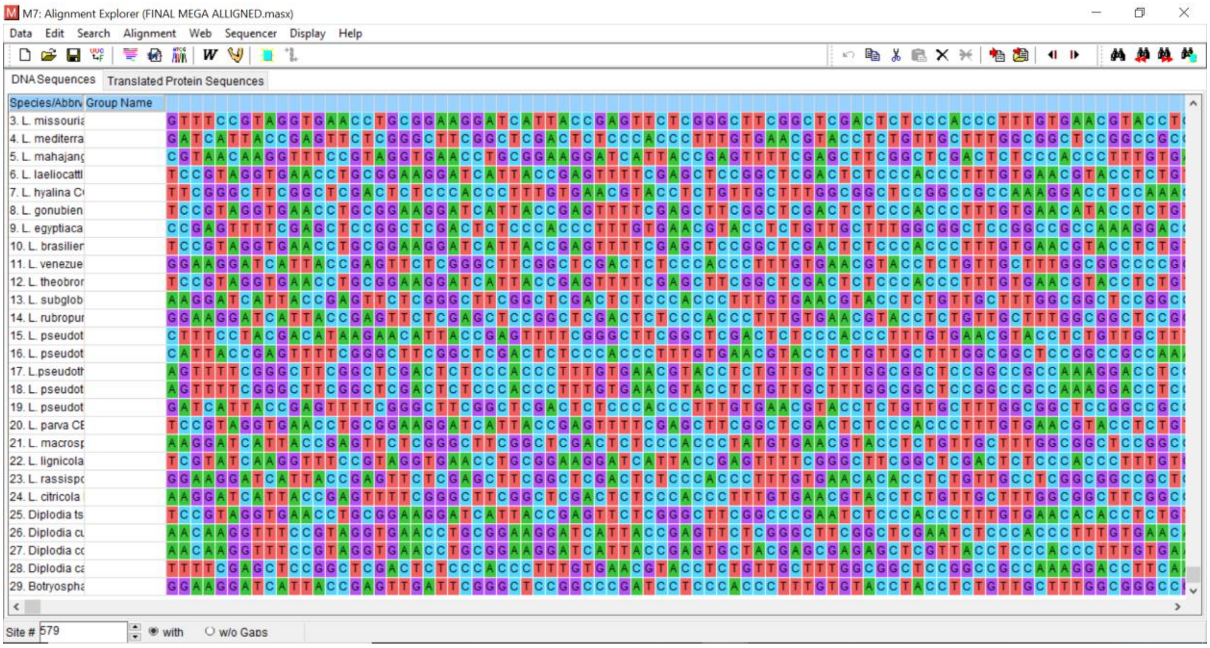
M7: Alignment Explorer (FINAL MEGA ALLIGNED.masx)
Data Edit Search Alignment Web Sequencer Display Help
DNA Sequences
Translated Protein Sequences
Species/Abbrv
Group Name
3. L. missouria
4. L. mediterra
5. L. mahajanç
6. L. laeliocattl
7. L. hyalina C
8. L. gonubien
9. L. egyptiaca
10. L. brasilier
11. L. venezue
12. L. theobror
13. L. subglob
14. L. rubropur
15. L. pseudot
16. L. pseudot
17. L.pseudoth
18. L. pseudot
19. L. pseudot
20. L. parva CE
21. L. macrosp
22. L. lignicola
23. L. rassispc
24. L. citricola
25. Diplodia ts
26. Diplodia cu
27. Diplodia cc
28. Diplodia ca
29. Botryospha
Site # 579
with
w/o Gaps

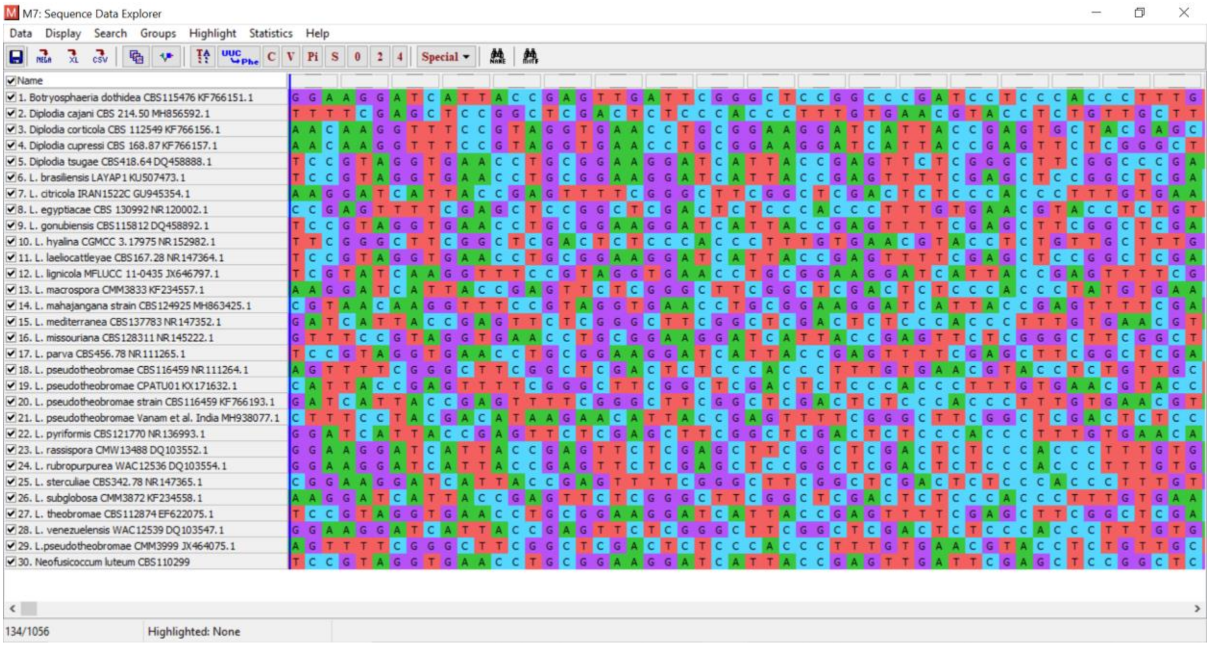
M7: Sequence Data Explorer
Data Display Search Groups Highlight Statistics Help
C V Pi S 0 2 4 Special
Name
1. Botryosphaeria dothidea CBS115476 KF766151.1
2. Diplodia cajani CBS 214.50 MH856592.1
3. Diplodia corticola CBS 112549 KF766156.1
4. Diplodia cupressi CBS 168.87 KF766157.1
5. Diplodia tsugae CBS418.64 DQ458888.1
6. L. brasiliensis LAYAP1 KU507473.1
7. L. citricola IRAN1522C GU945354.1
8. L. egyptiacae CBS 130992 NR120002.1
9. L. gonubiensis CBS115812 DQ458892.1
10. L. hyalina CGMCC 3.17975 NR152982.1
11. L. laeliocattleyae CBS167.28 NR147364.1
12. L. lignicola MFLUCC 11-0435 JX646797.1
13. L. macrospora CMM3833 KF234557.1
14. L. mahajangana strain CBS124925 MH863425.1
15. L. mediterranea CBS137783 NR147352.1
16. L. missouriana CBS128311 NR145222.1
17. L. parva CBS456.78 NR111265.1
18. L. pseudotheobromae CBS116459 NR111264.1
19. L. pseudotheobromae CPATU01 KX171632.1
20. L. pseudotheobromae strain CBS116459 KF766193.1
21. L. pseudotheobromae Vanam et al. India MH938077.1
22. L. pyriformis CBS121770 NR136993.1
23. L. rassispora CMW13488 DQ103552.1
24. L. rubropurpurea WAC12536 DQ103554.1
25. L. sterculiae CBS342.78 NR147365.1
26. L. subglobosa CMM3872 KF234558.1
27. L. theobromae CBS112874 EF622075.1
28. L. venezuelensis WAC12539 DQ103547.1
29. L.pseudotheobromae CMM3999 JX464075.1
30. Neofusicoccum luteum CBS110299
134/1056
Highlighted: None

Phylogenetic reconstruction:

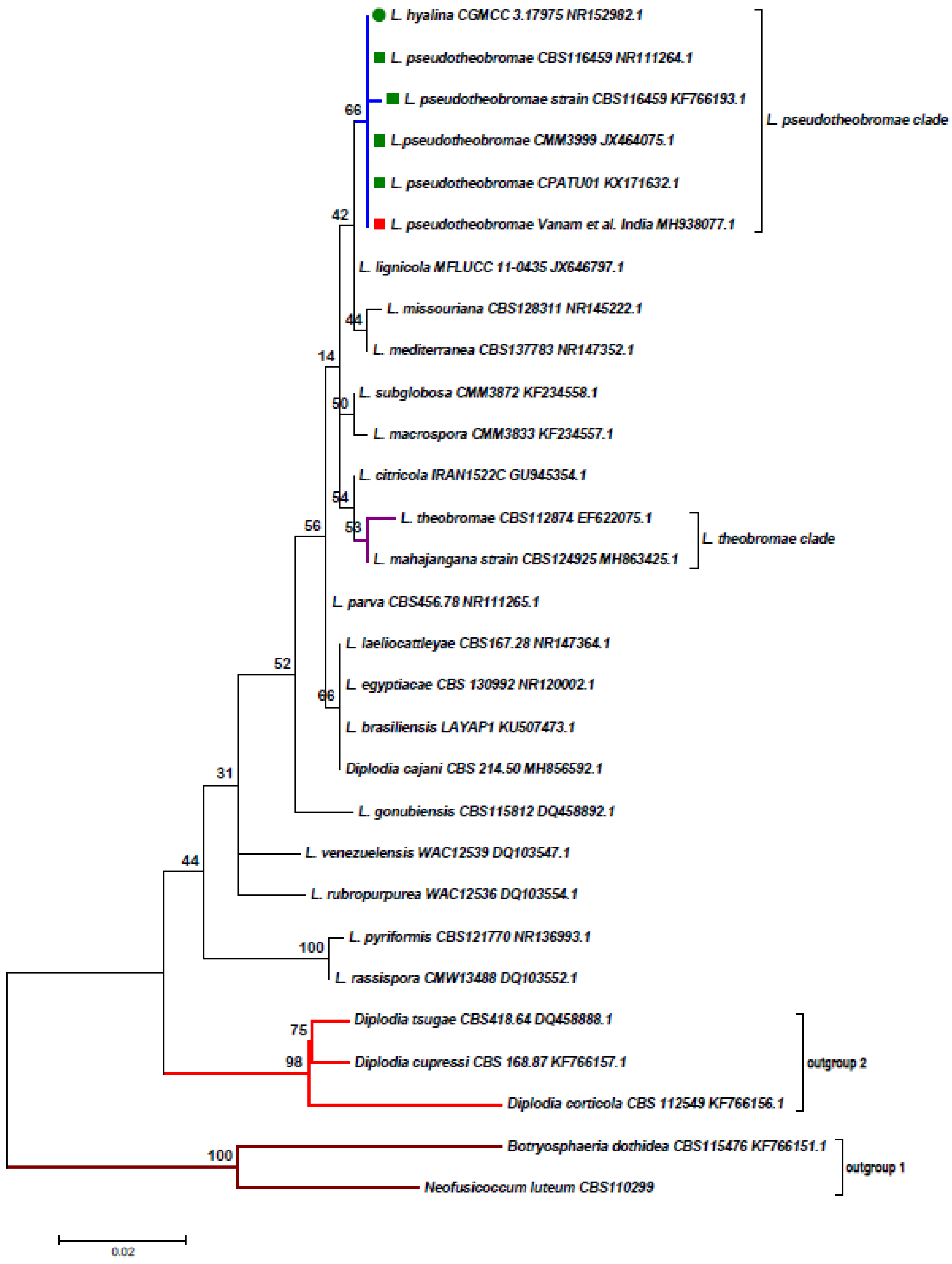

Supplement: Supplementary File 1 [file acmi-1-055-s001.pdf]
